# Endemic and epidemic *Acinetobacter baumannii* clones: a twelve-year study in a tertiary care hospital

**DOI:** 10.1186/s12866-015-0383-y

**Published:** 2015-02-25

**Authors:** Pilar Villalón, Sylvia Valdezate, Teresa Cabezas, Montserrat Ortega, Noelia Garrido, Ana Vindel, María J Medina-Pascual, Juan A Saez-Nieto

**Affiliations:** Laboratorio de Taxonomía, Servicio de Bacteriología, Centro Nacional de Microbiología, Instituto de Salud Carlos III, Majadahonda-Pozuelo km2, 28220 Madrid, Majadahonda Spain; Laboratorio de Biotecnología Hospital de Poniente El Ejido, Almería, Spain; Laboratorio de Infecciones Intrahospitalarias Servicio de Bacteriología, Centro Nacional de Microbiología Instituto de Salud Carlos III, Majadahonda Madrid, Spain

**Keywords:** *A. baumannii*, PFGE, Sequence type, MLVA, Clone

## Abstract

**Background:**

Nosocomial outbreaks of multidrug-resistant *Acinetobacter baumannii* are of worldwide concern. Using pulsed-field gel electrophoresis (PFGE), multilocus sequence typing (MLST), and multiple locus variable number tandem repeat sequence (VNTR) analysis (MLVA), the present work examines the genetic diversity of the endemic and epidemic *A. baumannii* clones isolated in a single hospital over a twelve-year period.

**Results:**

PFGE analysis of 405 *A. baumannii*-*calcoaceticus* complex isolates detected 15 *A. baumannii* endemic/epidemic PFGE types (EE1 to EE15) that grouped into five clusters: EE1-EE8, EE9, EE10, EE11 and EE12-EE15. The MLST sequence type (ST) distributions were: international clone II (ST-2) 60%, international clone III (ST-3) 26.7%, ST-15 6.7%, and ST-80 6.7%. MLVA-8_Orsay_ returned 17 allelic profiles. The large (L) VNTR marker profiles were fully concordant with the detected STs, and concordant with 14 up to 15 PFGE types. Imipenem resistance was detected in five PFGE types; the prevalence of the *bla*_OXA-58-like_ and *bla*_OXA-40-like_ genes was 60% and 40% respectively.

**Conclusions:**

PFGE proved to be a vital tool for analysis of the temporal and spatial distribution of the clones. MLST and the VNTR L-markers grouped the isolates into clonal clusters. The wide diversity of MLVA small (S)-markers, however, did not permit clustering. The present results demonstrate the persistence of several endemic PFGE types in the hospital, the involvement of some of them in outbreaks, and the inter hospital transmission of extensively drug-resistant ST-15 and ST-80.

## Background

The bacterium *Acinetobacter baumannii* is very tolerant to desiccation and has developed resistance against many of the antimicrobial agents in common use [1]. Both these features render it ideally suited to survive in the hospital environment, and invest it with the power to cause important nosocomial outbreaks [2]. Multidrug-resistant (MDR) *A. baumannii* infection is a worldwide health problem that has a negative impact on the morbidity and the mortality of the affected patients [3,4]. The epidemiological study of MDR *A. baumannii* outbreaks is essential if they are to be ended and their recurrence prevented. Clonal analysis of the strains involved is a vital part of these studies [5]. This can be undertaken via genotyping techniques such as pulsed-field gel electrophoresis (PFGE) [6,7], multilocus sequence typing (MLST) [7-9], and multiple locus variable number tandem repeat sequence (VNTR) analysis (MLVA) [10-12]. Genotyping provides information concerning outbreaks; e.g., the genetic diversity of clones, their temporal and spatial distribution, their implication on endemic or epidemic events, the acquisition source of the bacteria, as well as the number of affected patients; that is basic for preventive and control measures to be implemented.

A previous study of *A. baumannii* isolates from Spanish hospitals [13] showed the predominance of international clone II (sequence type 2 [ST-2]). Two novel clones were also detected - ST-79 and ST-80 - as well as international clonal complex 1/ST-81. The minor clones detected included international clone III (ST-3), ST-15 and ST-32.

The aim of the present work was to study the endemic and epidemic clones of *A. baumannii* in a not previously studied single hospital over a long period, and to compare them, using the aforementioned genotyping methods, with other nosocomial clones circulating in Spain.

## Results

### PFGE analysis

PFGE analysis of the 405 *A. baumannii*-*calcoaceticus* complex isolates returned 120 different PFGE types. Among these, 105 PFGE types (covering 174 isolates) were considered sporadic given their wide genetic disparity and small number of isolates (<5). Fifteen PFGE types, however, covering 231 *A. baumannii* isolates, were considered endemic or epidemic, and selected for further study.

**Figure 1:**
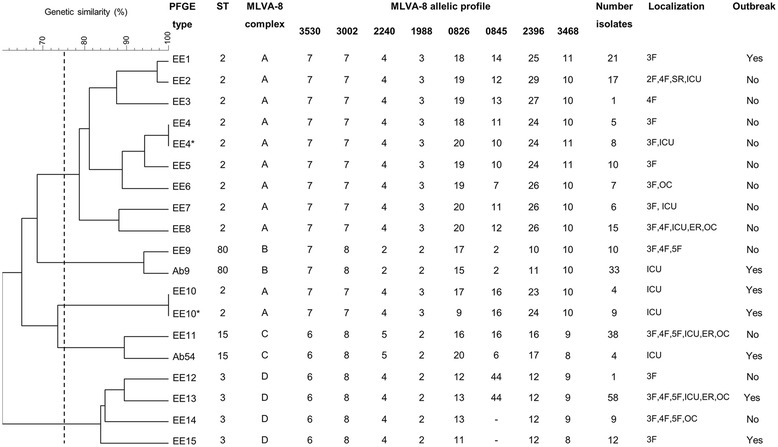
**Genetic diversity of endemic and epidemic** ***Acinetobacter baumannii*** **clones.** Left to right: PFGE genetic similarity dendogram, PFGE types, sequence types (ST), MLVA-8_Orsay_ complexes, repeat number for the eight VNTR markers, number of isolates, location in the hospital, and involvement in outbreaks. Pairs EE4-EE4* and EE10-EE10* had the same PFGE type but different MLVA type. The broken line in the dendogram shows the 75% genetic similarity cut-off. Abbreviations: 2 F, second floor; 3 F, third floor; 4 F, fourth floor; 5 F, fifth floor; ICU, intensive care unit; SR, surgery room; ER, emergency room; OC, out-patient clinic consultation room. Dash means lack of amplification. Ab9 and Ab54 are epidemic PFGE types detected in other hospitals.

**Figure 2:**
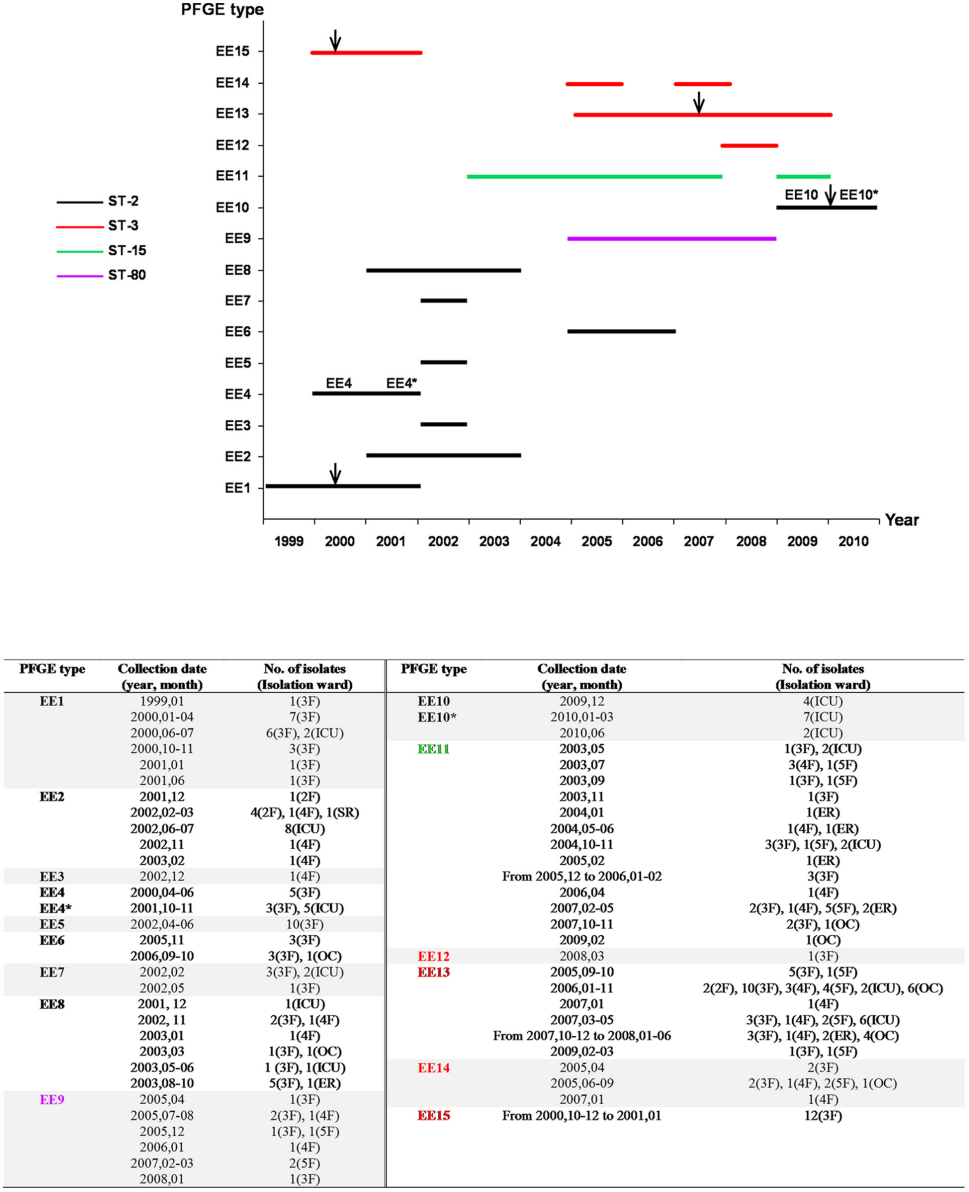
**Distribution of endemic and epidemic** ***Acinetobacter baumannii*** **PFGE types.** The top chart shows the temporal distribution of the 15 endemic and epidemic PFGE types, and the sequence types (ST). Vertical arrows indicate the nosocomial outbreaks. The bottom table shows the detailed data of the temporal and spatial distribution of the PFGE types. Abreviations: 2 F, second floor; 3 F, third floor; 4 F, fourth floor; 5 F, fifth floor; ICU, intensive care unit; SR, surgery room; ER, emergency room; OC; out-patient clinic consultation room.

Figure 1 shows the PFGE genetic similarity dendogram. The PFGE types were numbered EE1 to EE15. The Hunter-Gaston diversity index (HGDI) [14] calculated for the PFGE technique with respect to the further-examined 231 isolates was 0.880 (Table 1). The genetic similarity range for their 15 PFGE types was 60.4 – 100%. Five clusters (75% cut-off) were observed; their genetic similarity values were EE1-EE8 (78.8 – 100%), EE9-Ab9 (94.1%), EE10-EE10* (100%), EE11-Ab54 (89.7%), and EE12-EE15 (83.7 – 89.7%). Figure 2 shows the temporal and spatial distribution of the endemic/epidemic clones.

**Table 1:** **MLVA-8**_**Orsay**_ **data and comparison of PFGE, MLST and MLVA discriminatory power in endemic and epidemic** ***Acinetobacter baumannii*** **PFGE types**

**MLVA locus or Genotyping technique**	**Repeat size (bp)**	**No. of repeats mode (no. of repeats range)**	**Alleles or no. of genotypes**	**HGDI** ^***a***^	**CI (95%)** ^***b***^
Abaum_3530	60	6 (6 – 7)	2	0.502	(0.499, 0.508)
Abaum_3002	57	8 (7 – 8)	2	0.496	(0.482, 0.510)
Abaum_2240	99	4 (2 – 5)	3	0.345	(0.275, 0.415)
Abaum_1988	77-80	2 (2 – 3)	2	0.641	(0.617, 0.665)
Abaum_0826	9	13 (9 – 20)	9	0.833	(0.813, 0.853)
Abaum_0845	7	44 (2 – 44)	9	0.819	(0.795, 0.843)
Abaum_2396	6	12 (10 – 29)	9	0.807	(0.777, 0.837)
Abaum_3468	6	9 (8 – 11)	4	0.658	(0.625, 0.691)
PFGE			15	0.880	(0.858, 0.902)
MLST			4	0.655	(0.624, 0.686)
MLVA-8			17	0.883	(0.863, 0.903)

^*a*^Hunter-Gaston diversity index (HGDI) calculations were performed taking into account all 231 isolates in the studied 15 PFGE types, except for Abaum_0845 (N = 210), owing to the lack of amplification in EE14 and EE15.

^*b*^HGDI 95% confidence interval.

### *Acinetobacter baumannii* epidemic sequence types

For each gene, the number of alleles and polymorphic sites (in parenthesis) were: *cpn60* 4 (6), *fusA* 4 (3), *gltA* 3 (2), *pyrG* 2 (1), *recA* 3 (4), *rplB* 4 (3), and *rpoB* 4 (2). Four different STs were detected: ST-2, ST-3, ST-15 and ST-80. The HGDI for the MLST technique was 0.655 (Table 1).

International clone II (ST-2) involved clusters EE1-EE8 and EE10 (Figure 1), and included 60% of the studied PFGE types. International clone III (ST-3) involved cluster EE12-EE15 and 26.7% of the PFGE types. ST-15 and ST-80 were detected in clusters EE11 and EE9 respectively, both representing 6.7% of the PFGE types.

### *Acinetobacter baumannii* epidemic MLVA-8_Orsay_ types

**Figure 3:**
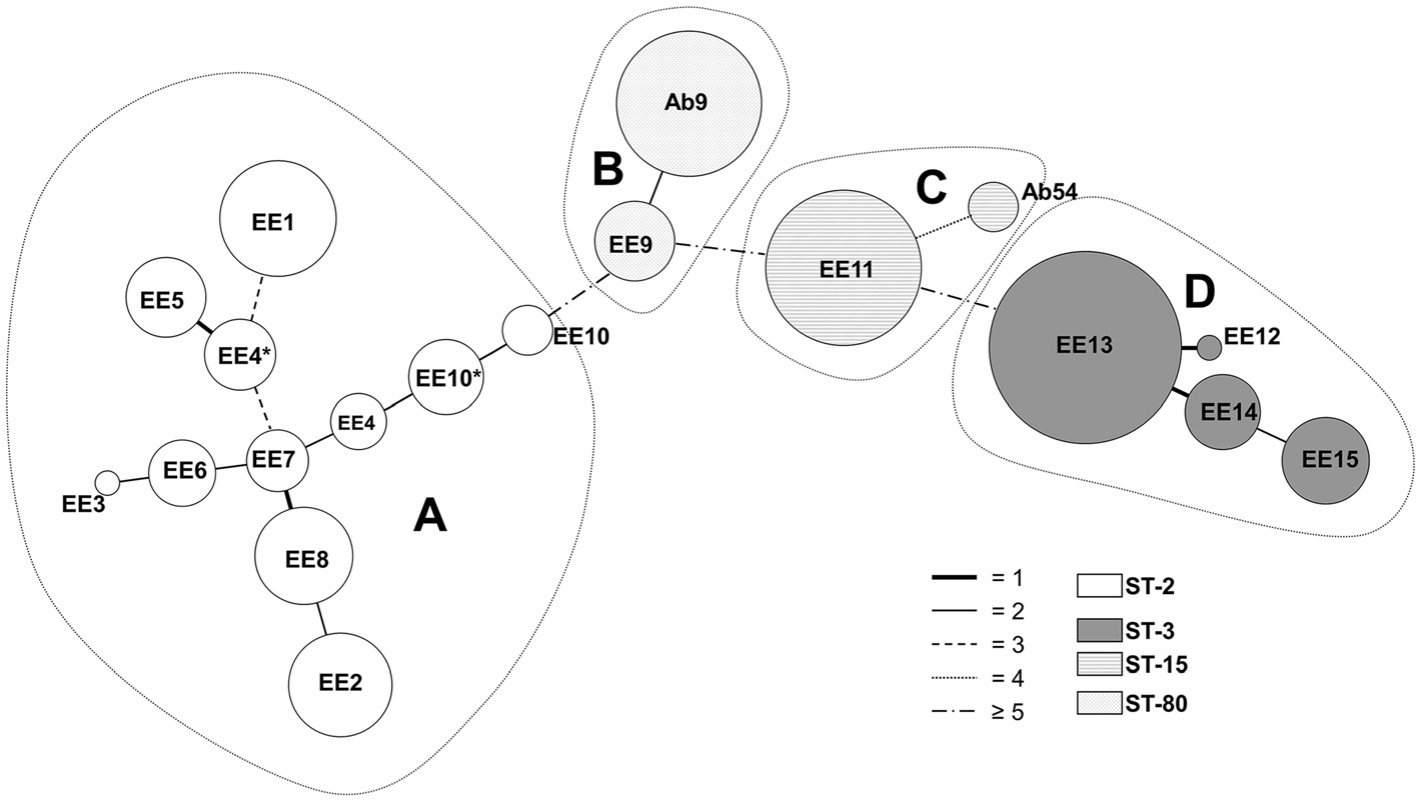
**Minimum spanning tree showing MLVA clustering.** Circles represent the different MLVA types, the size is proportional to the number of isolates. The corresponding PFGE types are indicated inside the circles, the color indicates the sequence type (ST). The connecting lines indicate the distance between MLVA types, coded according to the number of locus variants. Dotted lines group the four MLVA complexes A, B, C, and D.

Seventeen MLVA-8_Orsay_ allelic profiles were detected among the 15 studied PFGE types since the pairs EE4-EE4* and EE10-EE10* had different allelic profiles despite sharing the same PFGE type. The HGDI for the technique, taking into account all the VNTR loci examined, was 0.883. The individual HGDI scores for these markers ranged from 0.345 to 0.833. Microsatellites were more diverse (0.658 ≤ HGDI ≤ 0.833) than minisatellites (0.345 ≤ HGDI ≤ 0.641). Assuming a 40% cut-off for MLVA-8_Orsay_ genetic similarity (data not shown), four MLVA complexes were identified: A, B, C, and D. Figure 1 and Table 1 show the MLVA-8_Orsay_ data. Figure 3 shows the minimum spanning tree for MLVA-8_Orsay_ clustering.

Amplification products were obtained for all VNTR markers except for Abaum_0845 in EE14 and EE15. A previously described, non-conserved 77–80 bp repeat unit in the Abaum_1988 locus [11] was detected in all PFGE types, instead of the 26 bp repeat unit described by Pourcel [10].

### Susceptibility to antimicrobial agents, β-lactamase genes, and insertion sequences

The antimicrobial susceptibility rates recorded were 100% for colistin, 82.4% for sulbactam, 64.7% for imipenem, 64.7% for meropenem, 35.3% for amikacin, 35.3% for minocycline, 17.6% for doxycycline, and 17.6% for trimethoprim-sulfamethoxazole. All isolates showed resistance to mezlocillin, piperacillin, piperacillin-tazobactam, cefotaxime, ceftazidime, cefepime, gentamicin, tobramycin, tetracycline, ciprofloxacin, and levofloxacin.

All 15 PFGE types had an extensively drug-resistant (XDR) phenotype involving non-susceptibility to ≥1 agent in all but ≤2 antimicrobial categories [15]. Resistance to imipenem (MIC ≥4 μg/ml) was detected in five of the PFGE types: EE1, EE9, EE10, EE11, and EE15.

Carbapenem hydrolyzing oxacillinase (CHO) genes and metallobetalactamase (MBL) genes were sought in the five PFGE types resistant to imipenem, as well as in Ab9 and Ab54 (Table 2). EE1, EE11, EE15 and Ab54 carried the CHO gene *bla*_OXA-58-like_, with IS*Aba2* and IS*Aba3* upstream and downstream respectively. EE9, EE10, and Ab9 carried the *bla*_OXA-40-like_ gene. No MBL genes were amplified. *Acinetobacter*-derived cephalosporinase (ADC) and OXA-51 genes were also sought among the PFGE types with ST-80 or ST-15, and compared to those of Ab9 and Ab54 (which respectively show these STs). Pairs EE11-Ab54 and EE9-Ab9 had the same pattern of ADC, OXA-51 and CHOs genes and associated insertion sequences (IS) (Table 2).

**Table 2:** **β-lactamase genes and associated insertion sequences in** ***A. baumannii*** **with an imipenem MIC of >32 μg/ml**

**PFGE type**	**MLST sequence type**	***Acinetobacter*** **-derived cephalosporinase type**	***OXA*** **-51 type**	**Acquired carbapenem hydrolyzing oxacillinase**	**Metallo beta-lactamase**
EE1	2	Not typed	Not typed	IS*Aba2*−*OXA*-58−IS*Aba3* ^*f*^	ND^*g*^
EE10	2	Not typed	Not typed	*OXA*-40	ND
EE10^*a^	2	Not typed	Not typed	*OXA*-40	ND
EE15	3	Not typed	Not typed	IS*Aba2*−*OXA*-58−IS*Aba3*	ND
EE9	80	IS*Aba1*−*ADC*-1^*c*^	*OXA*-64/99/132^*d*^	*OXA*-40	ND
Ab9^*b*^	80	IS*Aba1*−*ADC*-1	*OXA*-64/99/132	*OXA*-40	ND
EE11	15	*ADC*-2	IS*Aba1*−*OXA*-64/99/132^*e*^	IS*Aba2*−*OXA*-58−IS*Aba3*	ND
Ab54^*b*^	15	*ADC*-2	IS*Aba1*−*OXA*-64/99/132	IS*Aba2*−*OXA*-58−IS*Aba3*	ND

^*a*^ EE10 and EE10* had the same PFGE type but different MLVA type.

^*b*^Ab9 and Ab54 were epidemic PFGE types detected in other hospitals.

^*c*^IS*Aba1* was detected upstream of *ADC*-1.

^*d*^The *OXA*-64/99/132 sequence was consistent with *OXA*-64, *OXA*-99 and *OXA*-132 [23].

^*e*^IS*Aba1* was detected upstream of *OXA*-64/99/132.

^*f*^IS*Aba2* and IS*Aba3* were detected upstream and downstream of *OXA*-58 respectively.

^*g*^ND: not detectable.

## Discussion

This study includes all *A. baumanni*-*calcoaceticus* complex isolates collected in a hospital during a twelve-year period. The clonal analysis of these isolates identified 15 endemic *A. baumannii* PFGE types, four of which were also epidemic because were involved in nosocomial outbreaks. Three *A. baumannii* nosocomial outbreaks occurred in the studied hospital between 1999 and 2010 (Figures 1 and 2). The first was in 1999–2000 in the pneumology area on the hospital’s third floor. This affected 20 patients and involved two PFGE types: EE1 and EE15. Both, EE1 and EE15 were first detected during this outbreak and persisted endemically till 2001 on the third floor. The second outbreak was in the ICU in March 2007, with four patients affected. This was caused by PFGE type EE13. This same PFGE type was found to be endemic between 2005 and 2009 on the third, fourth, and fifth floors of the hospital, and in the ICU, the emergency room, and out-patient consultation rooms. The third outbreak, from December 2009 to March 2010, also involved the ICU and was caused by PFGE type EE10; four patients were affected. The remaining 11 PFGE types (EE2-EE9, EE11, EE12, and EE14) were responsible for no epidemic event, but were found to be endemic; *i. e*. they were isolated on several occasions in different parts of the hospital environment. The temporal distribution of the 15 PFGE types was variable; it showed a range from one to six years, highlighting the long persistence of EE9, EE13 and EE11 during four, five and six years, respectively. The spatial distribution of the 15 PFGE types was universal in the hospital, although third floor (113 isolates) and ICU (44 isolates) were the most affected wards, probably due to the kind of activity and patients cared in these areas. All but three PFGE types -EE2, EE3, and EE10- were detected in the third floor, and eight out of fifteen PFGE types were detected in the ICU. The most ubiquitous PFGE types were EE8, EE11 and EE13. Long persistence, wide distribution and antimicrobial resistance are risk factors for new outbreaks. Figure 2 shows the temporal and spatial distribution of the 15 endemic/epidemic PFGE types.

The MLST results correlated with PFGE clustering. Most of the PFGE types (60%; clusters EE1-EE8 and EE10) circulating in the hospital during the study period were of sequence type ST-2 (or international clone II [9]). The majority presence of this clone is usual for Spanish hospitals [13], as in other Mediterranean countries [16], and indeed other parts of the world [17,18]. A previous study [13] showed that the highly prevalent ST-2 was detected on PFGE types that had very different DNA band patterns. This diversity is also detected in this study with the presence of ST-2 in clusters EE1-EE8 and EE10 -which showed only 65% genetic similarity-. ST-3 (or international clone III [9], PFGE cluster EE12-EE15), has been involved in many nosocomial outbreaks around the world [19], and in the present study was the second most common within PFGE types (26.7%). ST-15 (PFGE cluster EE11) and ST-80 (PFGE cluster EE9) were the two least common (6.7%), although both have been described as involved in outbreaks in other Spanish hospitals. International clonal complex1/ST-81, and sequence types ST-79 and ST-32 were not detected in the present study, but all are important nosocomial clones previously detected in Spanish hospitals [13].

MLVA-8_Orsay_ returned a perfect correlation between the L marker allelic profiles and the ST results. Therefore, MLVA-8_Orsay_ L marker results correlate with PFGE clustering. The same L marker allelic profiles (and therefore STs) were even seen for certain PFGE types in different clusters, e.g., L markers 7-7-4-3 (ST-2) in clusters EE1-EE8 and EE10. The S marker allelic profiles showed a wide diversity. As an example, the same PFGE type can show different S marker allelic profiles over short periods [10]. For instance, between 2000 and 2001, a change in three S loci was recorded in PFGE type EE4 (recorded as types EE4 and EE4*; Figures 1, 2 and 3). Similarly, over the space of just a few months between 2009 and 2010 - within the same outbreak - changes in two S loci were recorded in PFGE type EE10 (recorded as EE10 and EE10*; Figures 1, 2 and 3). In one of the latter loci (allele 0826), the change involved a remarkable descent in the number of tandem repeats from 17 (in EE10) to 9 (in EE10*). In fact, the diversity associated with allele 0826 has led some authors [11] to exclude it in the establishment of MLVA complexes. In the present study, the variability associated with the S markers was so great that it was not possible to define criteria for establishing MLVA complexes, neither to apply criteria defined by other authors [10,11]. The MLVA complexes were therefore defined assuming a 40% genetic similarity cut-off, and full correlation was also seen between the MLST and MLVA-8_Orsay_ complexes, i.e., ST-2 with A, ST-80 with B, ST-15 with C, and ST-3 with D (Figures 1 and 3).

MLVA-8_Orsay_ L markers are very useful for establishing phylogenetic relationships because of their stability [10], and reveal relationships similar to those provided by MLST analysis [12,20]. To date, several criteria for the formation of MLVA complexes using L and S markers have been proposed for *A. baumannii* [10,11]. Agreement has been achieved with the interpretation of L markers but no consensus has been reached with respect to S markers, −something already achieved for other pathogens [21]- specifically with the maximum number of allele and repeat differences that can be accepted to cluster different MLVA types into the same MLVA complex.

The 15 studied PFGE types all showed XDR. Indeed, three (EE1, EE10 and EE15) of the four PFGE types involved in the three outbreaks described in the present work, were also resistant to imipenem. Resistance is common in *A. baumannii* nosocomial outbreaks since resistant strains are selected for in hospital environments, especially in high risk areas such as ICUs [4,22]. Other PFGE types resistant to imipenem, endemic but not involved in any outbreak, were detected in the present study, such as EE9 and EE11. Both showed a wide distribution across several areas of the hospital, and were isolated over long time periods (Figures 1 and 2). EE9 was isolated from 2005 to 2008 on the third, fourth, and fifth floors, and EE11 was isolated over 2003–2007 and during 2009 from the same floors as EE9, in addition to the ICU, emergency room, and out-patient clinic consultation rooms.

The susceptibility and β-lactamase results fitted well with the epidemiological data for Spain, where the resistance of *A. baumannii* to carbapenems is basically due to the activity of OXA-40 and OXA-58 [23]. OXA-23 activity has been commonly reported around the world, but only recently reported for *A. baumannii* in Spain [24]. The idea that interhospital transmission of XDR *A. baumannii* clones occurs is reinforced by the detection of a common pattern for the ADC, OXA-51, and CHOs genes, and their associated ISs, for example as seen in PFGE type pairs EE11 and Ab54, and EE9 and Ab9.

## Conclusions

The comparison of the three genotyping methods shows that MLST is very useful in global *A. baumannii* clonal studies, clustering the isolates into big clones. Though laborious, PFGE provides essential information in local studies of *A. baumannii* outbreaks. Currently, MLVA-8_Orsay_ L markers provide similar information than MLST, but it has the drawback that no consensus has been reached regarding the interpretation of S markers. MLVA-8_Orsay_ could be a better typing method than PFGE for *A. baumannii* if new S markers were added to MLVA panel and agreed criteria for MLVA clustering were established.

In the present study, the most prevalent was international clone II (ST-2), followed by international clone III (ST-3), ST-15 and ST-80. The period 1999–2004 was characterized by the predominant presence of international clone II. The period 2005–2010, however, saw diversification, with the increasing presence of international clone III (ST-3), ST-15 and ST-80. Extensively drug-resistance was universal and the genes providing resistance to carbapenems were *bla*_OXA-58-like_ and *bla*_OXA-40-like_. The finding of the same β–lactamase pattern in PFGE type pairs EE9 and Ab9, and EE11 and Ab54, suggests the interhospital transmission of the XDR *A. baumannii* clones ST-80 and ST-15. Finally, this study highlights that the endemic presence of extremely resistant PFGE types poses a potential risk for future outbreaks [25]. The present results also confirm the persistence of fifteen endemic PFGE types in a tertiary care hospital and the involvement of four of them in three outbreaks.

## Methods

### PFGE-typed strains and study design

The bacteria examined were 405 not previously studied *A. baumannii*-*calcoaceticus* complex isolates from a 285-bed public tertiary care hospital in the southeast of Spain; all were collected over the period 1999–2010 (both sporadically and during epidemic situations) and were sent to the Spanish National Center for Microbiology for their molecular characterization. Table 3 shows the distribution by year, sample type, and hospital ward of isolation for all 405 isolates.

**Table 3:** **Distribution of the 405** ***Acinetobacter baumannii-calcoaceticus*** **complex isolates recovered over the twelve-year study period**

Year^*a*^	1999 (1/7)	2000 (28/52)	2001 (14/18)	2002 (35/50)	2003 (22/37)	2004 (9/35)
	2005 (30/35)	2006 (35/46)	2007 (32/50)	2008 (9/23)	2009 (7/26)	2010 (9/26)
Type of sample^*a,b*^	Respiratory (73/152)	Wound (39/69)	Environmental^*c*^ (52/55)	Exudates colonization screening (34/51)		
	Urine (14/32)	Blood (11/20)	Not informed (0/17)	Catheter tip (5/5)	Other clinical samples (3/4)	
Hospital ward^*a,b*^	3rd Floor: IM^*d*^ (115/181)	ICU (44/53)				
	4th Floor: IM, and General and Digestive Surgery (21/49)	Out-patient consultation rooms (15/41)				
	5th Floor: IM (22/38)	Emergency Room (9/19)				
	Not informed (2/18)	1st Floor-Pediatrics-Gynecology (0/2)				
	2nd Floor: Orthopedic Surgery-Mental Health (2/2)	Surgery Room (1/1)				
	Hemodialysis (0/1)					

^*a*^In parenthesis are indicated the number of endemic-epidemic *A. baumannii* isolates in the total number of *A. baumannii-calcoaceticus* complex isolates.

^*b*^Distributions are ordered from most to least frequent in relation to the total number of isolates.

^*c*^Types of environmental samples (N=55): not informed (n=16), nursing trolley (10), bed (6), door (4), telephone (4), electric panel (3), breathing equipment (3), monitor (3), closet (3), oxygen equipment (2), table (1).

^*d*^IM: Internal Medicine.

The clinical samples were taken as part of standard patient care and also for this purpose, the bacterial strains were sent to a public national reference laboratory for their clonal analysis. This study focused on bacteria and no identifiable human data were used, therefore ethical approval was exempted.

All the isolates were analyzed by PFGE. All distinguishable PFGE patterns were interpreted as different PFGE types. Clustering was determined by using the unweighted-pair group method with arithmetic averages (UPGMA) and by using Dice’s coefficient. The tolerance was set at 0.8% and the optimization at 1.5%. All calculations were performed by using InfoQuest software (Applied Maths, Saint-Marten-Latem, Belgium). The PFGE types that were suspected of being endemic or epidemic were selected for further study. The selection criterion was based on the number of isolates and the genetic similarity; i.e., PFGE types that had ≥5 isolates, and PFGE types that although had <5 isolates showed ≥85% genetic similarity with the former PFGE types. A cut-off of 75% was subsequently chosen for clustering the selected PFGE types into big clones. The member isolates (N = 231) were identified as *A. baumannii* by *bla*_OXA-51-like_ gene amplification [26], analyzed by MLST and MLVA, tested for antimicrobial susceptibility, and examined for β-lactamase genes and their commonly associated ISs. The origin of the selected isolates was environmental (n = 52) and clinical (n = 179, one isolate per patient).

### MLST and MLVA

MLST analysis was performed following the protocol of the Institute Pasteur (www.pasteur.fr/recherche/genopole/PF8/mlst/Abaumannii.html). The *cpn60*, *fus*A, *glt*A, *pyr*G, *rec*A, *rpl*B and *rpo*B genes were amplified using the PCR conditions described by Bartual [8], and then sequenced. The primers used to amplify the genes were those described in the above-mentioned protocol, except the primers used for *rpo*B, which were those reported in earlier work [13]. Each allelic profile was assigned a ST.

MLVA typing was performed using the MLVA-8_Orsay_ method [10], i.e., involving four L markers (Abaum_3530, Abaum_3002, Abaum_2240, and Abaum_1988) with large repeat units of ≥9 bp (minisatellites), and four S markers (Abaum_0826, Abaum_0845, Abaum_2396, and Abaum_3468) with small repeat units in the 2–8 bp range (microsatellites). For each VNTR locus, the alleles were sequenced and the length of the repeat and number of repetitions analyzed using the Tandem Repeats Finder tool (http://tandem.bu.edu/trf/trf.html). The number of repeats was calculated by rounding up according to Vergnaud [21]. MLVA clustering was represented using a minimum spanning tree produced using InfoQuest software (Applied Maths, Saint-Martens-Latem, Belgium).

The comparison of the discriminatory power of the PFGE, MLST, and MLVA typing techniques was done calculating the Hunter-Gaston diversity index (HGDI) [14] with a 95% confidence interval [27]. The MLVA polymorphism index was calculated for individual and combined VNTR loci.

### Antimicrobial susceptibility, β-lactamase genes and insertion sequences

Susceptibility testing was performed using the MicroScan NM31 microdilution method (Dade Behring, West Sacramento, CA, USA). Disc diffusion was used to determine susceptibility to doxycycline, minocycline and colistin (all from Oxoid, Basingstoke, Hants, UK). Imipenem susceptibility was confirmed using E-test strips (bioMérieux, Marcy-l’Etoile, France). Results were interpreted using the Clinical Laboratory Standards Institute criteria for *Acinetobacter* spp. The control strains used were *E. coli* ATCC 25922 and *E. coli* ATCC 35218.

Screening for the ADC genes [28], and the four CHO genes [29] *bla*_OXA-51-like_, *bla*_OXA-23-like_, *bla*_OXA-40-like_, and *bla*_OXA-58-like_, as well as their most commonly associated ISs (IS*Aba1*, IS*Aba2*, IS*Aba3*, IS*Aba4* and IS*18*), was performed. Screening was also undertaken for the MBL genes [30] *bla*_IMP_, *bla*_VIM_, *bla*_SIM-1_, *bla*_GIM-1_, *bla*_SPM-1_ and *bla*_NDM-1_ [31]. The PCR methods used were those described in previous work [23].

### Sequencing analysis

Purification of the PCR products was performed using the illustra™ ExoProStar™ 1-Step Kit (GE Healthcare, NJ, USA). Sequencing was performed in a 3730XL sequencer using the BigDye Terminator Cycle Sequencing Kit v.3.1 (Applied Biosystems, Foster City, CA, USA). The sequences were assembled using Lasergene SeqMan II software (DNA Star, Inc., Madison, Wis., USA).

### Interhospital comparison of PFGE types

Two epidemic PFGE types, Ab54 and Ab9, that had been previously described from other Spanish hospitals [13], were added to the study. In 2003, Ab54 caused an outbreak in the intensive care unit (ICU) of a hospital in the Province of Almería (where the studied hospital is also located). In 2005, Ab9 was involved in two outbreaks in the ICUs of two different hospitals in the same southwestern province of Spain (Badajoz). Ab54 was included to the study for comparison with EE11 since both shared ST-15. Ab9 was included for comparison with EE9 since both share ST-80. The interhospital comparison of these four PFGE types included PFGE, MLST, and MLVA typing; as well as the analysis of ADC, OXA-51, CHO, MBL genes and associated ISs.

## Abbreviations

PFGE: Pulsed-field gel electrophoresis
MLST: Multilocus sequence typing
VNTR: Variable number tandem repeat
MLVA: Multiple locus variable number tandem repeat sequence (VNTR) analysis
ST: Sequence type
L: Large
S: Small
MDR: Multidrug-resistant
XDR: Extensivelydrug-resistant
HGDI: Hunter-Gaston diversity index
MIC: Minimum inhibitory concentration
CHO: Carbapenem hydrolyzing oxacillinase
MBL: Metallobetalactamase
ADC: *Acinetobacter*-derived cephalosporinase
IS: Insertion sequence
2 F: Second floor
3 F: Third floor
4 F: Fourth floor
5 F: Fifth floor
ICU: Intensive care unit
SR: Surgery room
ER: Emergency room
OC: Out-patient clinic consultation room
